# Roles of IGFBP-3 in cell migration and growth in an endophytic tongue squamous cell carcinoma cell line

**DOI:** 10.1038/s41598-022-15737-y

**Published:** 2022-07-07

**Authors:** Esther Feng Ying Ng, Atsushi Kaida, Hitomi Nojima, Masahiko Miura

**Affiliations:** Department of Oral Radiation Oncology, Graduate School of Medical and Dental Sciences, Tokyo Medical & Dental University, 1-5-45 Yushima, Bunkyo-ku, Tokyo, 113-8549 Japan

**Keywords:** Oral cancer, Cell growth, Cell migration, Growth factor signalling, Time-lapse imaging

## Abstract

Insulin-like growth factor binding protein-3 (IGFBP-3) is a member of the IGFBP family that has high affinity for IGFs and functions as either an oncogene or tumor suppressor in various types of cancer. We previously found that *IGFBP3* mRNA levels are higher in endophytic-type human tongue squamous cell carcinoma (TSCC) that is more invasive and more prone to metastasis than exophytic and superficial types. This finding prompted us to investigate the roles of IGFBP-3 in TSCC using SAS cells, which were originally derived from endophytic-type TSCC. Specifically, we used SAS cells that express a fluorescent ubiquitination-based cell-cycle indicator (Fucci). RNA-sequencing analysis indicated that IGFBP-3 is associated with cell migration and cell growth. In fact, IGFBP-3 knockdown downregulates cell migration and causes cells to arrest in G_1_. This migratory potential appears to be cell cycle–independent. IGFBP-3 knockdown also reduced levels of secreted IGFBP-3; however, decreased migratory potential was not rescued by exogenous recombinant human IGFBP-3. Furthermore, ERK activity was downregulated by IGFBP-3 depletion, which suggests that MEK/ERK signaling may be involved in IGFBP-3-mediated cell migration. We therefore conclude that intracellular IGFBP-3 enhances cell migration independently of the cell cycle in TSCC with a higher metastatic potential.

## Introduction

Insulin-like growth factors (IGFs) circulate in the bloodstream and play important roles in tumor development, growth, and metastasis by interacting with IGF-I receptor (IGF-IR)^1–3^. Insulin-like growth factor binding protein-3 (IGFBP-3) is one of six members of the IGFBP family and has high affinity for IGF-I and -II^4,5^. Due to the secretory signal peptide sequence in IGFBP-3, IGFBP-3 is found not only inside the cell but is also secreted into blood plasma^6^. Secreted IGFBP-3 can bind to IGFs and increase their half-life. Moreover, the binary complex of IGFBP-3 and IGFs can interact with the acid-labile subunit and form a ternary complex, leading to further stabilization of IGFs^7^. Therefore, IGFBP-3 may inhibit IGF-IR signaling by antagonizing IGFs, resulting in inhibition of cell survival and sensitization to chemotherapy^5,8^. Furthermore, IGFBP-3 exerts growth inhibitory effects by interacting with transforming growth factor-β (TGF-β) receptor and Bcl-2-associated X protein (BAX) independently of IGFs^9–13^.

Increasingly, however, studies have found evidence in support of an oncogenic role for IGFBP-3. High levels of IGFBP-3 expression have been detected in head and neck cancer^14^ and are correlated with poor clinical outcomes in breast cancer^15,16^. Exogenous IGFBP-3 is also known to promote cell migration by upregulating transcription of the gene that encodes vascular cell adhesion molecule 1 (VCAM-1) in osteosarcoma cells^17^. Thus, IGFBP-3 may act as either an oncogene or tumor suppressor depending on cellular context and tumor type.

Tongue squamous cell carcinoma (TSCC) is among the most common head and neck squamous cell carcinomas (HNSCCs) and is classified into three groups according to macroscopic appearance, namely superficial, exophytic, and endophytic. In general, endophytic-type TSCC is more invasive and more prone to metastasis than other types. Moreover, clinical outcomes in patients with endophytic-type TSCC are generally poor^18–21^. We have previously identified 26 genes that are overexpressed specifically in endophytic-type TSCC, compared with other types, by gene expression microarray analysis using clinical biopsy samples. Among the genes identified*, PARVB* expression was clinically correlated with increased frequency of cervical lymph node metastasis and decreased overall survival. We also found *PARVB* knockdown inhibits cell migration in a TSCC cell line, SAS^22^. This cell line was originally derived from a patient with endophytic-type TSCC who subsequently developed cervical lymph node metastasis, distant metastasis to the lung, and died^23^. Notably, in the same study, *IGFBP3* was at the top of the identified 26 gene list in terms of statistical significance^22^. We therefore pursued the role of IGFBP-3 using the SAS cell line.

The fluorescent ubiquitination-based cell-cycle indicator (Fucci) allows us to determine the cell cycle position of any cell based on its fluorescent color^24^. Fucci is useful for analyzing various phenomena regarding cell cycle-associated events at the single-cell level^25,26^. We previously established a SAS cell line expressing the Fucci system, which we designated as SAS-Fucci cells^27^. In this study, we employed SAS-Fucci cells to determine if IGFBP-3 functions in cell migration and growth in TSCC with a highly malignant phenotype, and to determine if the migration and growth phenotypes observed are cell cycle–dependent.

## Results

### IGFBP-3 knockdown leads to an altered gene expression profile in SAS-Fucci cells

**Figure 1:**
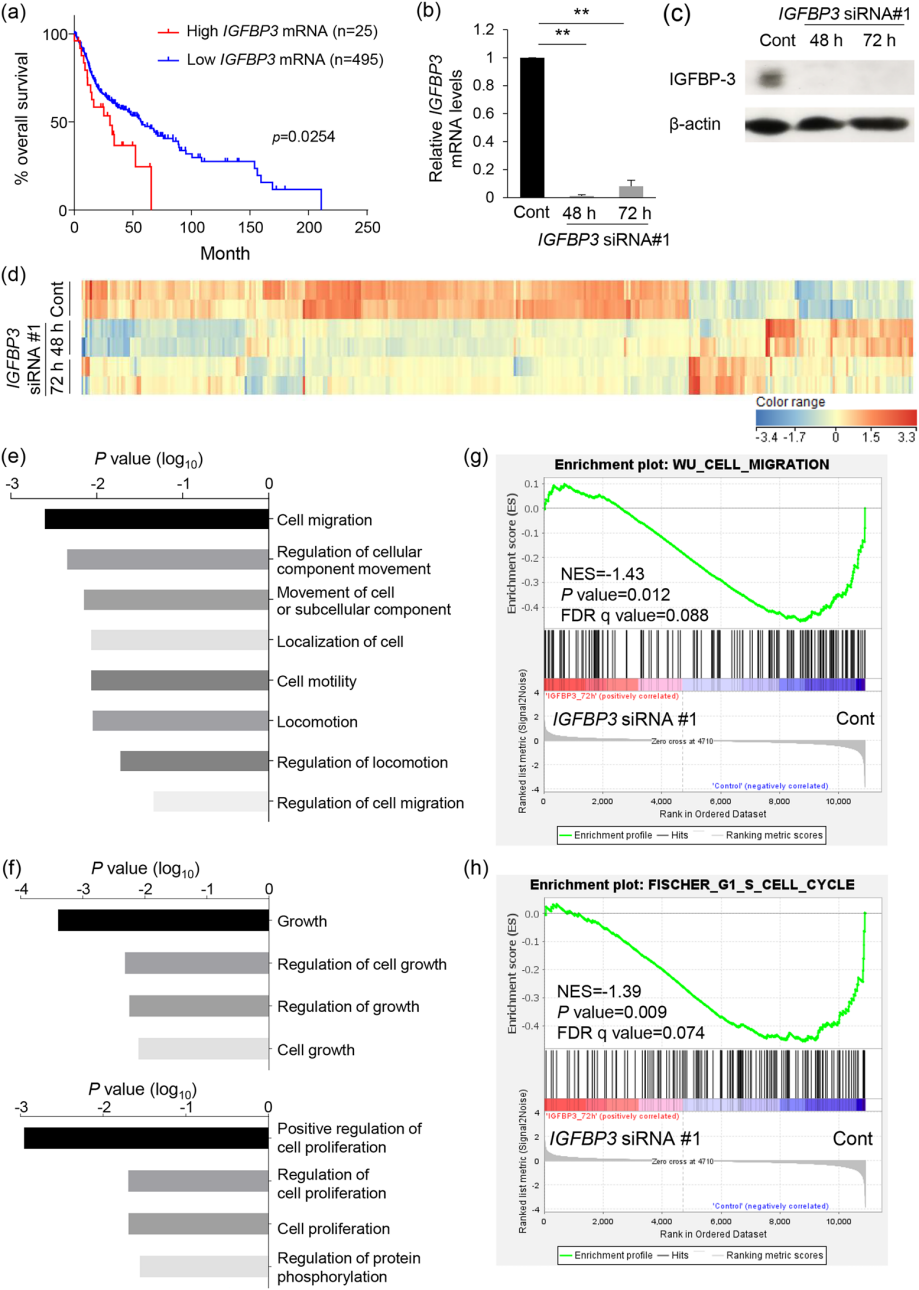
Changes in gene expression profile following IGFBP-3 knockdown in SAS-Fucci cells. (**a**) TCGA data-based Kaplan–Meier analyses for overall survival in patients with head and neck squamous cell carcinoma with either high (*n* = 25) or low (*n* = 495) levels of *IGFBP3* mRNA. A cohort is separated according to *Z*-scores relative to diploid samples. *p*-value; log-rank test. (**b**, **c**) *IGFBP3* mRNA levels by qPCR (**b**) and IGFBP-3 and β-actin protein levels by western blotting (**c**). Cells were transfected with either non-targeted control (Cont) or *IGFBP3* siRNA #1 (48 h or 72 h). Total mRNA or cell lysate was extracted either 48 h or 72 h after siRNA treatment. Means ± S.D. (*n* = 3 independent experiments). Uncropped blots for (**c**) are presented in Supplementary Fig. S7. (**d**) Hierarchical cluster analysis of differentially expressed genes between control (Cont) and IGFBP-3-knockdown (*IGFBP3* siRNA #1, 48 or 72 h) cells using the RNA-seq data (*n* = 2 per each group). Red and blue colors on the heat map represent up- and downregulation, respectively, of the indicated gene. (**e**, **f**) GO analysis using the gene set significantly downregulated by IGFBP-3 knockdown in RNA-Seq results from control and *IGFBP3* knockdown cells at 72 h. (**g**, **h**) GSEA of cell migration (**g**) and G_1_/S phase transition (**h**) signatures between control (Cont) and IGFBP-3 knockdown (*IGFBP3* siRNA #1) cells at 72 h. NES, normalized enrichment score; FDR, false discovery rate. **p < 0.01; one-way ANOVA with Tukey’s multiple comparison test (**b**).

The IGFBP-3 expression level has been implicated in clinical prognosis for various cancer types^14–16^. Furthermore, clinical outcomes are poor in patients with endophytic-type TSCC compared with patients with other types of TSCC^28,29^; however, the clinical significance of IGFBP-3 remains unclear for TSCC. Therefore, we determined the clinical correlation between *IGFBP3* expression levels and survival in patients with HNSCC using the TCGA database. *IGFBP3* mRNA levels were classified into two groups, namely high (*Z*-score > 2) and low (*Z*-score ≤ 2), according to the *Z*-scores relative to diploid samples. Overall survival was significantly lower in patients with high *IGFBP3* mRNA levels, as compared with patients with low levels (Fig. 1a). The small number of cases showing high IGFBP-3 expression may be due to a low proportion of endophytic TSCC in the cohort. This result supports our hypothesis that *IGFBP3* exhibits oncogenic behavior in HNSCC.

The biological functions of IGFBP-3 in TSCC remain to be elucidated. We knocked down IGFBP-3 using siRNA in human TSCC SAS-Fucci cells (Fig. 1b,c) and performed RNA sequencing (RNA-Seq) to determine the effect of IGFBP-3 knockdown on the gene expression profile. Hierarchical cluster analysis revealed that the expression of many genes was affected at both 48 and 72 h after IGFBP-3 knockdown (Fig. 1d). The gene ontology (GO) analysis result showed that genes significantly downregulated by IGFBP-3 knockdown are involved in biological processes related to cell migration and cell growth (Fig. 1e,f). Moreover, gene set enrichment analysis (GSEA) revealed that IGFBP-3 expression correlates negatively with gene sets related to cell migration and the G_1_/S transition: WU_CELL_MIGRATION (normalized enrichment score (NES) =  − 1.43, *p* value = 0.012, FDR q-value = 0.088) and FISCHER_G1_S_CELL_CYCLE (NES =  − 1.39, *p* value = 0.009, FDR q value = 0.074) (Fig. 1g,h). The RNA-Seq results suggest that IGFBP-3 expression is associated with both cell migration and cell-cycle progression in SAS-Fucci cells.

### Cell migration is inhibited by IGFBP-3 knockdown

**Figure 2:**
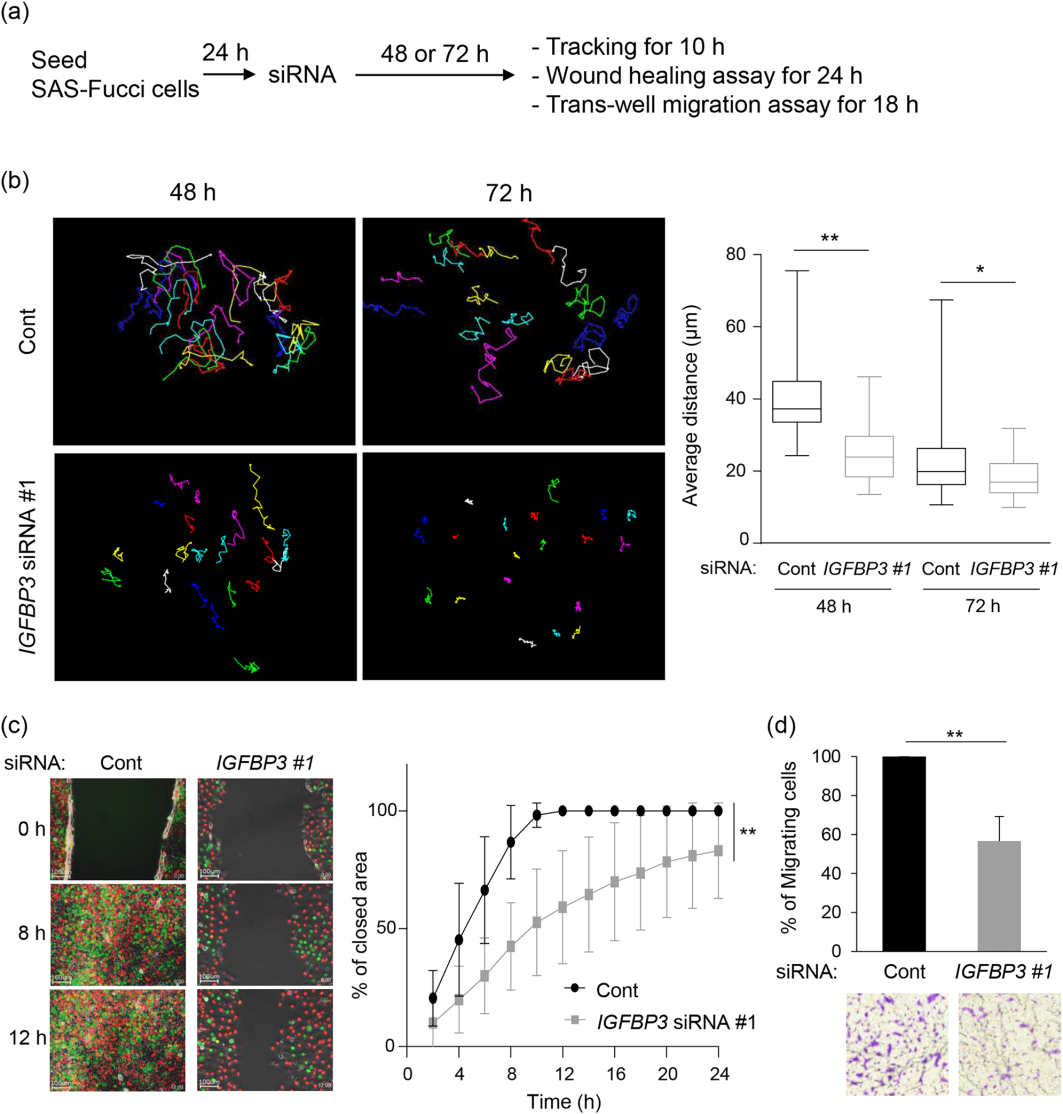
Reduced cell migration by IGFBP-3 knockdown in multiple assays. (**a**) Experimental flow to analyze cell migration. Twenty-four hours after cells were seeded, they were transfected with siRNA for either 48 or 72 h before the indicated experiments were performed. (**b**) Representative images of single-cell tracking during 10 h-observation (left) and quantification of average distance (right) during 10 h using SAS-Fucci cells 48 and 72 h after transfection with either non-targeted control (Cont) or *IGFBP3* siRNA #1. Individual colored lines indicate each cell track traced by the center of the nucleus during the observation period. Each average distance is represented as a box and whisker plot showing outliers, distribution intervals, interquartile range (box), and median. Three independent experiments were performed and representatives are shown. (**c**) Representative images (left) and quantitative analysis (right) of wound healing assays using non-target control (Cont) and IGFBP-3 knockdown (*IGFBP3 #1*) cells. Images were acquired by time-lapse imaging and time after wounding is shown. Means ± S.D. (*n* = 3 independent experiments). Significant difference was detected at all the indicated timepoints except at 2 h. (**d**) Quantitative analysis (top) and representative images (bottom) of trans-well migration assays using non-target control (Cont) and IGFBP-3-knockdown (*IGFBP3 #1*) cells (8 × 10^4^ cells). Migrating cells in the entire field were counted 18 h later. Means ± S.D. (*n* = 3 independent experiments). *p < 0.05, **p < 0.01; Kruskal–Wallis test with Dunn’s multiple comparisons test (**b**), two-way ANOVA with Sidak’s multiple comparisons test (**c**), or two-tailed Student’s t-test (**d**).

We previously reported that endophytic-type TSCC which is more invasive and more prone to metastasis expressed high levels of *IGFBP3*^22^. RNA-Seq results also suggest that IGFBP-3 is involved in cell migration (Fig. 1). Therefore, we first examined the effect of IGFBP-3 expression on cell migration using various methods (Fig. 2a).

Under normal conditions, SAS-Fucci cells exhibit very high migration activity, which sometimes made it difficult to track individual cells since some cells would disappear from the observation field. Single-cell tracking analysis based on time-lapse imaging revealed that IGFBP-3 knockdown decreased the average distance traveled during the 10 h observation period compared with control cells (Fig. 2b). Intriguingly, the difference was more pronounced at 48 h than at 72 h (Fig. 2b), which may be due to cell confluency. Hence, we subsequently performed single-cell tracking analysis 48 h after siRNA transfection. As indicated by our single-cell tracking results, IGFBP-3 knockdown reduced migratory potential according to our wound healing assay and trans-well migration assay (Fig. 2c,d). Since siRNA can cause off-target effects, we also used a second *IGFBP3*-specific siRNA, namely siRNA #2, to control for such effects. IGFBP-3 knockdown in SAS-Fucci cells by siRNA #2 impacted cell migration in a similar manner to siRNA #1 (Supplementary Fig. S1a-c). Moreover, to determine if these phenomena are observed in other TSCC cells with high metastatic potential, we used HSC-3 cells that express Fucci (HSC3-Fucci cells). HSC-3 cells were originally derived from metastatic lymph nodes of human TSCC patients and have been shown to have high metastatic potential^30^. IGFBP-3 knockdown by both *IGFBP3*-specific siRNAs #1 and #2 reduced cell migration in HSC3-Fucci cells as well (Supplementary Fig. S1d,e). Hence, IGFBP-3 appears to function in cell migration among human TSCC cell lines with high metastatic potential.

### Cell migration modulated by IGFBP-3 is independent of cell cycle

**Figure 3:**
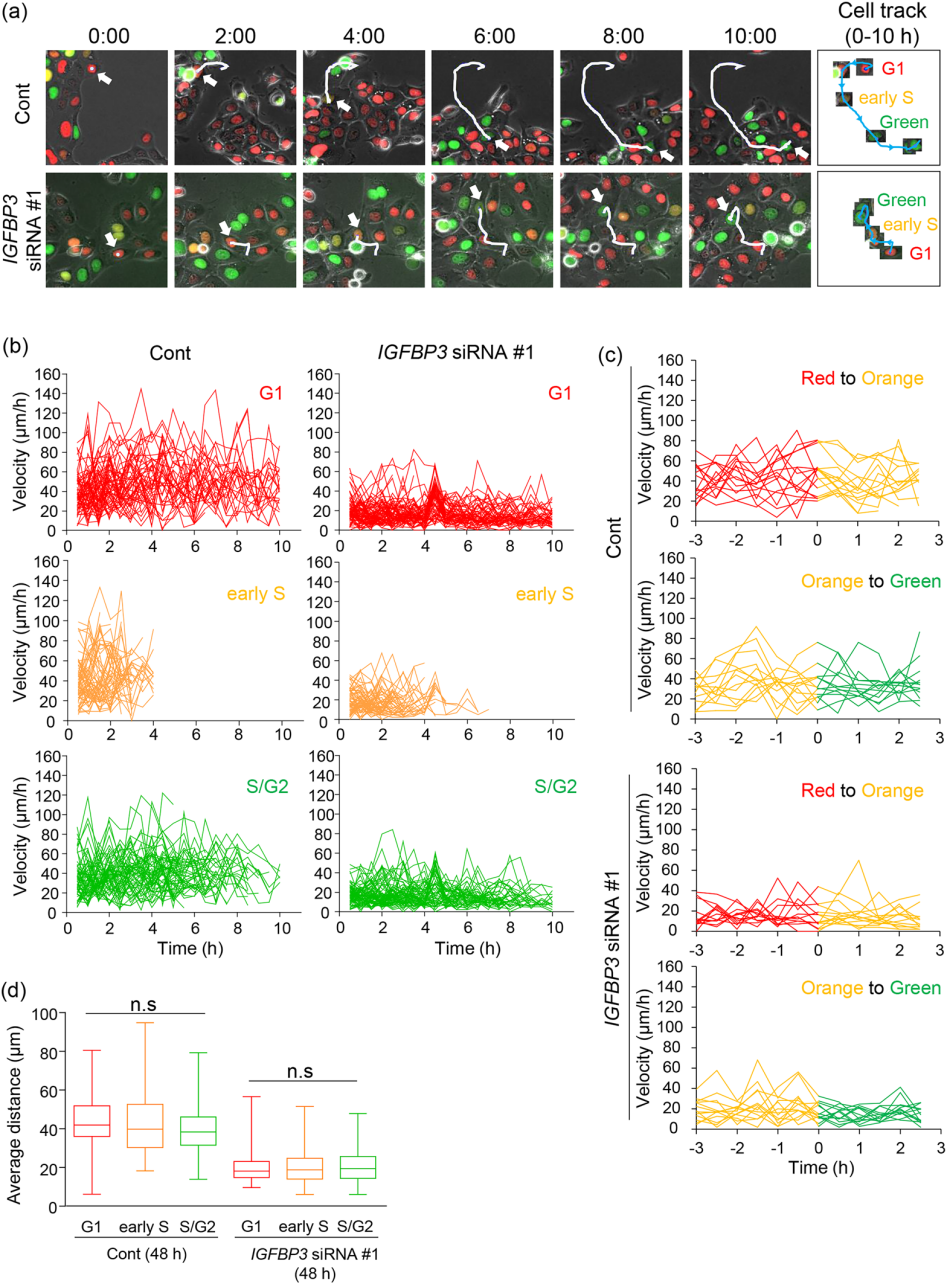
Non-significant effect of cell cycle on cell migration by IGFBP-3 knockdown. (**a**) Representatives of single-cell tracking during 10-h observation merged with fluorescent images in non-target control (Cont) and IGFBP-3 knockdown (*IGFBP3* siRNA #1) cells. Red, orange, and green cells are in G_1_, early S, and S/G_2_/M phases, respectively. Rounded green cells are in mitosis. White arrows and lines indicate the position of the traced cell at the indicated timepoints and a cell track from the start of the observation to the indicated timepoints, respectively. The cell traced at each timepoint is cropped and presented in the panel on the right. The cell track during the observation is shown as a blue line. Time elapsed after the start of observation is shown (hours: minutes). (**b**) Quantification of the change in velocity of each cell in G_1_, early S, and S/G_2_ phases using non-target control (Cont) and IGFBP-3 knockdown (*IGFBP3* siRNA #1) cells. Each line indicates the change in velocity of a single cell. Velocity was calculated based on distance traveled over 30 min. Individual lines for 62–73 cells are shown as representatives. (**c**) Quantification of the change in velocity of each cell during the transition from red to orange (upper, Red to Orange) or from orange to green (lower, Orange to Green) using non-target control (Cont) and IGFBP-3 knockdown (*IGFBP3* siRNA #1) cells. Individual lines of 13–15 cells are shown as representatives. T = 0 h is represented as the timing of transition. (**d**) Quantification of average distance of control and IGFBP3 knockdown cells during the observation in each cell-cycle phase. Cells were classified as being in either G_1_, early S, or S/G_2_ according to morphology and fluorescent color. Each average distance is represented as a box and whisker plot showing outliers, distribution intervals, interquartile range (box), and the median. Cell numbers of each group are 89–162 cells (**d**). Three independent experiments were performed, and representatives are shown. n.s. (not significant); Kruskal–Wallis test with Dunn’s multiple comparisons test (**d**).

The Fucci reporter system allows us to determine which phase of the cell cycle a cell is in, based on the fluorescent color that it generates^24^. Using this system, we could distinguish between cells in G_1_ (red), early S (orange), or S/G_2_ (green) phases to determine if cell-cycle position affects migratory potential (Fig. 3a).

First, we quantified the change in the velocity of single cells by measuring the distance traveled every 30 min. Overall, the velocity of each cell was similar between cell-cycle phases for both control and IGFBP-3 knockdown cells; however, the range of velocities varied considerably throughout the observation period, particularly among control cells (Fig. 3b). Additionally, we focused on the timing of the transition from red to orange (G_1_ to early S phase) and from orange to green (early S to S/G_2_ phase) for each cell (Fig. 3c). The transition duration did not significantly affect velocity in either control or IGFBP-3-knockdown cells (Fig. 3c). We also measured the average distance traveled by cells in G_1_, early S, and S/G_2_ phases (Fig. 3d), and found no significant difference. Hence, our velocity and distance measurements were in agreement. IGFBP-3 knockdown also reduced the average distance traveled by cells in all cell-cycle phases (Fig. 3d). These findings suggest that IGFBP-3 promotes cell migration irrespective of cell-cycle position.

### Secreted IGFBP-3 has minimal impact on cell migration

**Figure 4:**
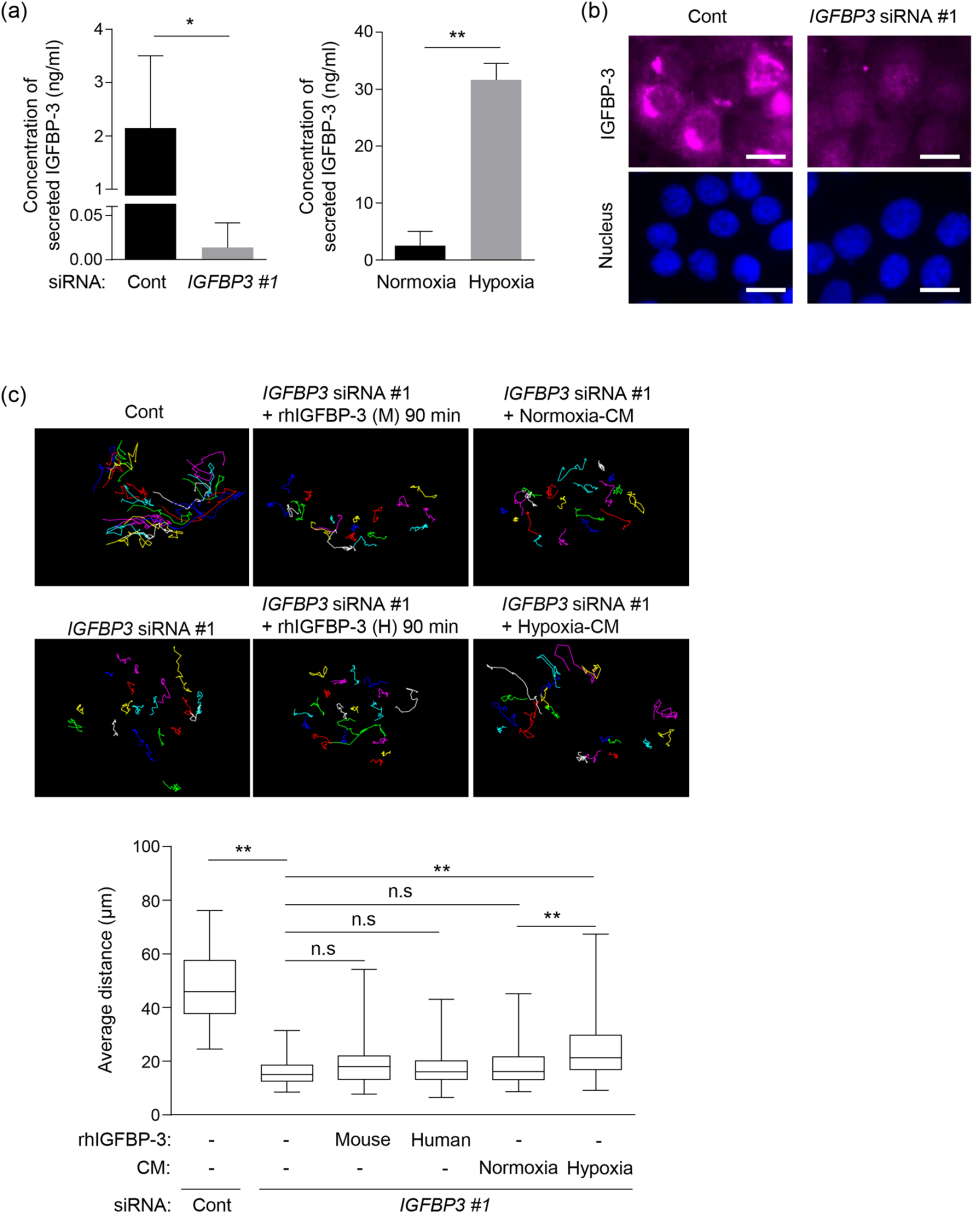
Minimal impact of secreted IGFBP-3 on cell migration. (**a**) Concentration of secreted IGFBP-3 determined by ELISA. SAS-Fucci cells were transfected with non-targeted control (Cont) or *IGFBP3* siRNA #1 for 48 h (left) and treated under either normoxia or hypoxia (right) for 24 h. After each treatment, conditioned media were collected and applied for ELISA. Means ± S.D. (*n* = 3 independent experiments). (**b**) Representative anti-IGFBP-3 immunofluorescence images of control (Cont) and IGFBP-3 knockdown (*IGFBP3* siRNA #1) cells. Scale bar: 10 μm. (**c**) Representative images of individual cell tracks (top) and average distance traveled (bottom) by IGFBP-3 knockdown (*IGFBP3* siRNA #1) and control (Cont) cells with or without recombinant human IGFBP-3 (rhIGFBP-3) derived from mouse or human cells and normoxic- or hypoxic-conditioned medium (CM). rhIGFBP-3 or CM was added 48 h after siRNA treatment, with which cells were pre-treated for 90 min before time-lapse imaging. Each colored line indicates individual cell tracks during the observation period. Each average distance is represented as a box-whisker plot showing outliers, distribution intervals, interquartile range (box), and median. Cell numbers in each group ranged between 80 and 160. Either two or three independent experiments were performed, and representatives are shown in (**b**, **c**). *p < 0.05, **p < 0.01; two-tailed Student’s *t*-test (**a**) or Kruskal–Wallis test with Dunn’s multiple comparisons test (**c**).

Since the IGFBP-3 protein possesses a secretory signal peptide sequence, it is secreted extracellularly^6^. We measured the concentration of IGFBP-3 in the culture medium by ELISA in order to determine the effect of IGFBP-3 knockdown on its secretion. The levels of IGFBP-3 secreted by IGFBP-3-knockdown cells were significantly lower compared with control cells (Fig. 4a-left). Similarly, IGFBP-3 knockdown significantly reduced intracellular IGFBP-3 levels (Fig. 4b), consistent with our western blotting results (Fig. 1b). Hypoxia has been shown to increase IGFBP-3 levels by upregulating HIF-1α transcriptional activity^31,32^. We observed that hypoxic SAS-Fucci cells secreted significantly more IGFBP-3 compared with normoxic cells (Fig. 4a-right).

To determine if secreted IGFBP-3 affects the migratory potential of SAS-Fucci cells, we applied two recombinant human IGFBP-3 proteins (rhIGFBP-3) of different origins, mouse and human, reconstituted with a carrier to IGFBP-3-knockdown cells 90 min and 24 h before time-lapse imaging and quantified the average distance traveled. Decreased migratory potential caused by IGFBP-3 depletion was not rescued by both rhIGFBP-3 irrespective of pre-incubation time (Fig. 4c and Supplementary Fig. S2). This result suggests that extracellular IGFBP-3 does not affect cell migration significantly in SAS-Fucci cells. Intriguingly, hypoxic-conditioned medium partially restored migratory potential while normoxic-conditioned medium did not (Fig. 4c). Although the concentration of IGFBP-3 in hypoxic-conditioned medium was lower than that of rhIGFBP-3 (100 ng/mL) used in this experiment, we observed higher migratory potential in IGFBP-3 knockdown cells, which suggests that other hypoxia-induced factors may contribute to the recovery of cell migration.

### ERK and AKT signaling modified by IGFBP-3 depletion is associated with cell migration

**Figure 5:**
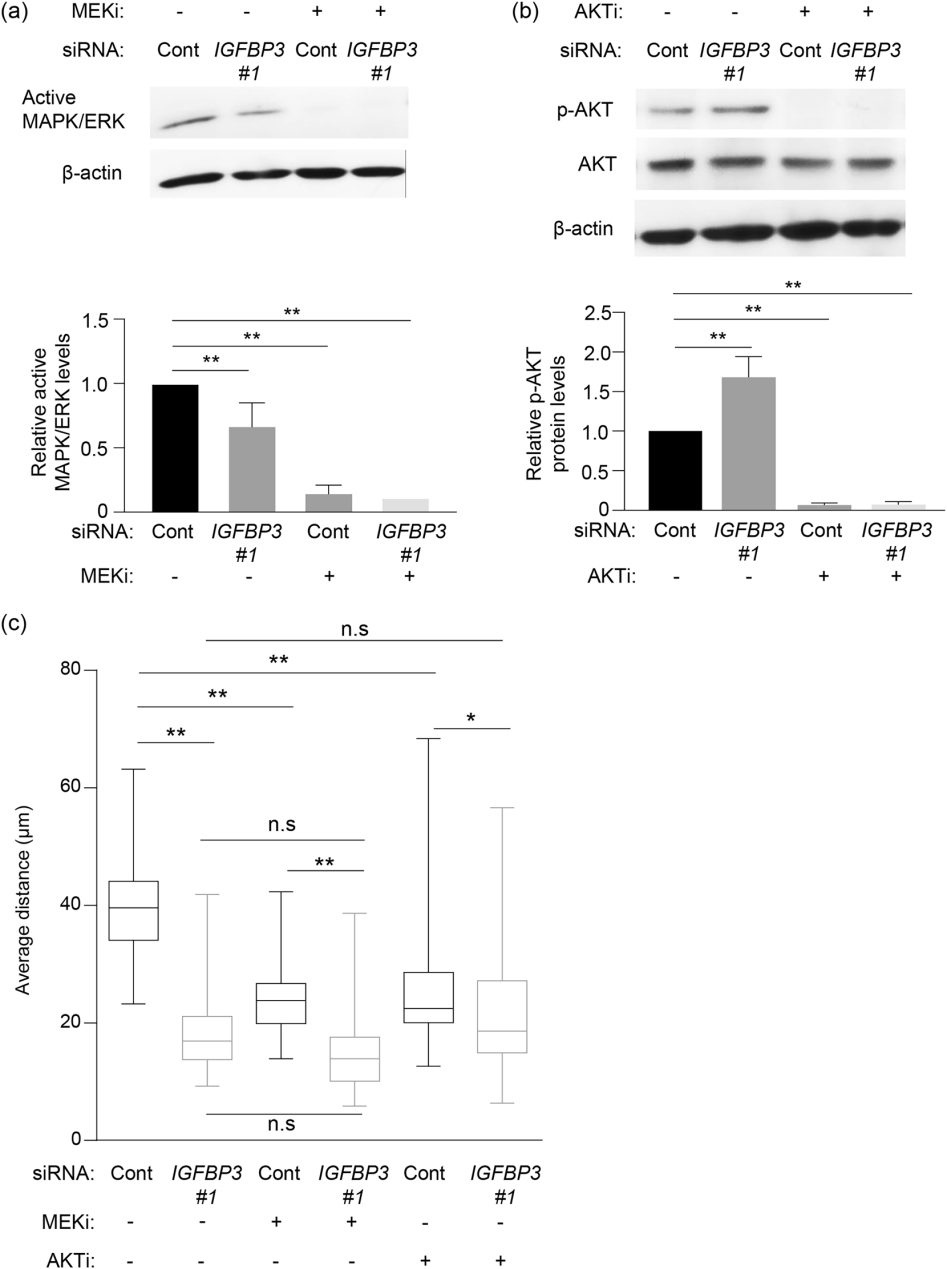
Association of ERK and AKT signaling modified by IGFBP-3 depletion with cell migration. (**a**, **b**) Western blotting (top) for active MAPK/ERK, phosphorylated AKT (p-AKT), AKT, and β-actin, and relative levels of active MAPK/ERK and p-AKT in control (Cont) and IGFBP-3 knockdown (IGFBP3 #1) cells either treated with MEK (MEKi; PD98059, 20 μM, (**a**) and AKT (AKTi; MK-2206, 5 μM, (**b**) inhibitors or left untreated. Cells were incubated with siRNA for 48 h. MEK and AKT inhibitors were added 90 and 30 min before cell lysates were extracted, respectively. Uncropped blots are presented in Supplementary Fig. S7. Active MAPK/ERK and p-AKT protein levels were normalized to β-actin and total AKT protein levels, respectively. Means ± S.D. (*n* = 3 independent experiments). (**c**) Average distance traveled by control (Cont) and IGFBP-3 knockdown (IGFBP3) cells with or without MEK (MEKi) or AKT (AKTi) inhibitor. Each average distance is represented as a box and whisker plot showing outliers, distribution intervals, interquartile range (box), and median. Each group contains 80 cells. Two or three independent experiments were performed. *p < 0.05, **p < 0.01; one-way ANOVA with Tukey’s multiple comparison test (**a**, **b**) or Kruskal–Wallis test with Dunn’s multiple comparisons test (**c**).

Migratory potential is upregulated by the activation of PI3K and MAPK signaling^33–38^. Moreover, IGFBP-3 promotes cell migration by activating β1 integrin-ERK signaling in oral squamous cell carcinoma cells^39^. This finding motivated us to investigate the effect of ERK and AKT signaling on cell migration in our study. IGFBP-3 knockdown significantly decreased migratory potential with the significant reduction in the level of active ERK (Fig. 5a,c). MEK inhibitor treatment also reduced migratory potential in control cells, while decreased cell migration caused by IGFBP-3 depletion was not affected by MEK inhibitor treatment (Fig. 5c). This result suggests that IGFBP-3 is partially involved in activating ERK and regulating cell migration. Surprisingly, unlike ERK activity, the level of phosphorylated AKT was significantly upregulated following IGFBP-3 knockdown as compared with the control (Fig. 5b). To investigate the dependency of AKT activity on cell migration, we treated cells with an AKT inhibitor and analyzed their migratory potential. As observed with the MEK inhibitor, AKT inhibitor treatment reduced the extent of cell migration in control cells (Fig. 5c), indicating that AKT activity also regulates cell migration in SAS-Fucci cells. In IGFBP-3 knockdown cells, the AKT inhibitor did not affect migratory potential, while upregulation of AKT phosphorylation was significantly decreased (Fig. 5b,c). These findings suggest that the activation of both ERK and AKT is required to promote cell migration, and that IGFBP-3 contributes to the activation of ERK rather than AKT.

### IGFBP-3 knockdown induces cell-cycle arrest

**Figure 6:**
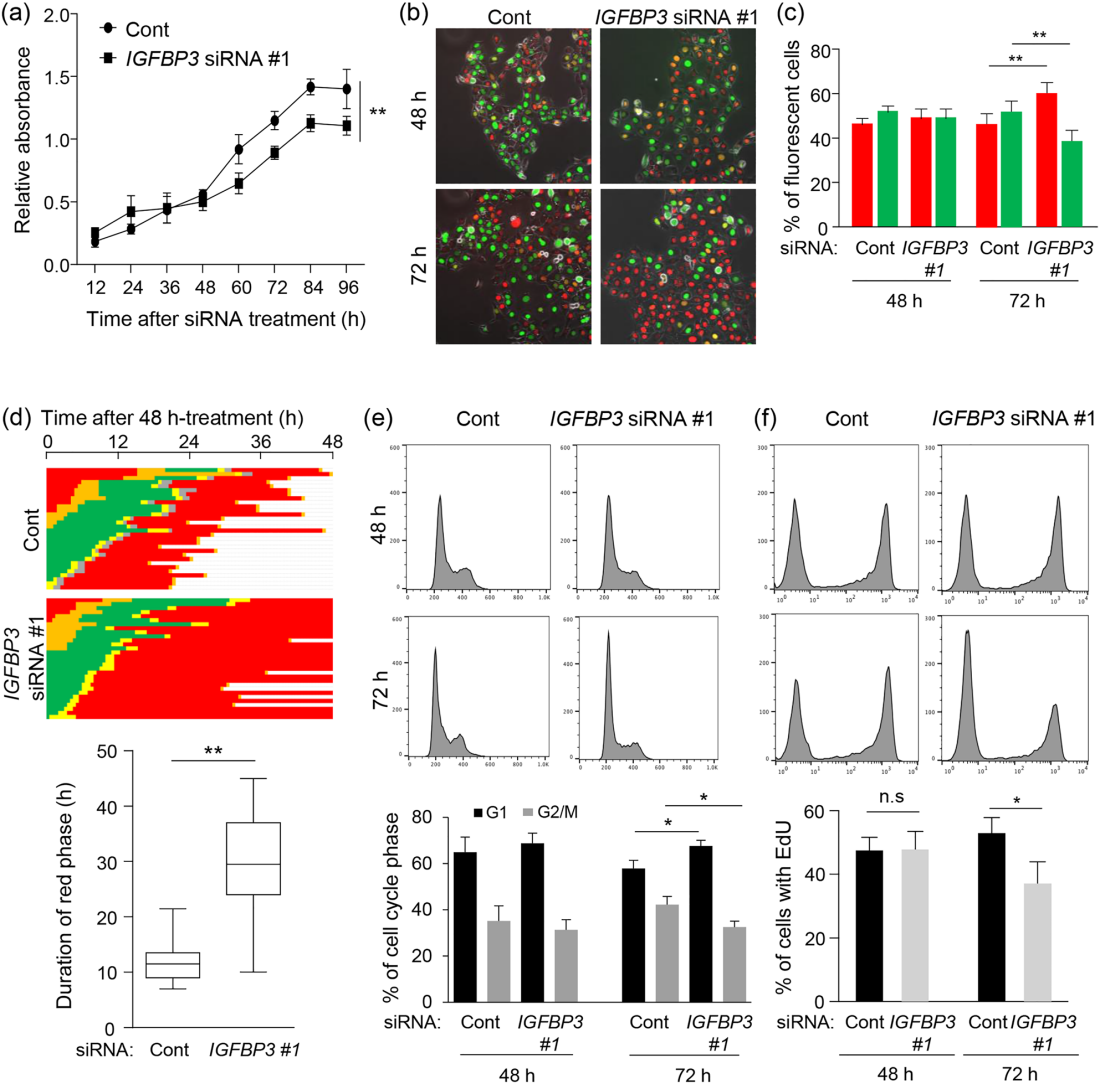
Inhibition of cell growth with prolongation of G1 phase by IGFBP-3 knockdown. (**a**) Quantitative analysis of cell proliferation assay using non-target control (Cont) and IGFBP-3 knockdown (*IGFBP3* siRNA #1) cells. Cell Counting Kit-8 assay was performed each time after siRNA treatment and relative absorbance was plotted. Means ± S.D. (*n* = 3 independent experiments). (**b**, **c**) Representative images and quantification of Fucci fluorescence in SAS-Fucci cells transfected with non-targeted control (Cont) and *IGFBP3* siRNA #1 at 48 (b: top) and 72 h (b: bottom). Means ± S.D. (*n* = 3 independent experiments). (**d**) Pedigrees (top) and duration of red phase (bottom) for SAS-Fucci cells transfected with either *IGFBP3* siRNA #1 or non-targeted control siRNA (Cont). Cells were traced between 0 and 48 h after siRNA treatment for 48 h. Red cells were sorted from the result of pedigree assay, and the duration of the red phase was measured from the start of the red phase to its end. Each duration is represented as a box and whisker plot showing outliers, distribution intervals, interquartile range, and median. (**e**, **f**) Representative histograms (upper) and quantification (bottom) of DNA content (**e**) and EdU incorporation (**f**) by FACS using SAS-Fucci cells transfected with either *IGFBP3* siRNA #1 or non-targeted control siRNA (Cont) at 48 and 72 h. Means ± S.D. (*n* = 3 independent experiments). *p < 0.05, **p < 0.01; two-way ANOVA with Sidak’s multiple comparisons test (**a**), one-way ANOVA with Tukey’s multiple comparison test (**c**, **e**, **f**), Mann–Whitney U-test (**d**).

In addition to the role of IGFBP-3 in cell migration, our RNA-Seq results indicate that IGFBP-3 is involved in cell growth in TSCC (Fig. 1). Several studies have also demonstrated that IGFBP-3 knockdown inhibits DNA synthesis and tumor growth^40,41^. Therefore, we wanted to determine the effect of IGFBP-3 on cell growth. Cell proliferation assay revealed that IGFBP-3 knockdown in SAS-Fucci cells significantly decreased proliferative potential, compared with control cells (Fig. 6a and Supplementary Fig. S3a), while there was no significant difference in cell proliferation between IGFBP-3 knockdown and control HSC3-Fucci cells (Supplementary Fig. S3b). This result suggests that the dependency of cell proliferation on IGFBP-3 might differ in cell lines. IGFBP-3 knockdown in SAS-Fucci cells significantly increased the fraction of red cells (i.e., cells in G_1_) but decreased the fraction of green cells (cells in S/G_2_ phase) only 72 h after siRNA treatment (Fig. 6b,c). Taking advantage of the Fucci, we also measured the duration of red phase (G_1_ phase) in each siRNA-treated cell. The duration of red phase in IGFBP-3 knockdown cells was longer than that in control cells (Fig. 6d). Based on DNA content, many IGFBP-3 knockdown cells accumulated in G_1_ only at 72 h (Fig. 6e), consistent with the observed Fucci fluorescence. Additionally, EdU incorporation, a DNA synthesis marker, was reduced in IGFBP-3 knockdown cells at 72 h (Fig. 6f). Taken together with our RNA-Seq results, these results suggest that IGFBP-3 also plays a role in cell growth, especially by regulating the G_1_/S transition, in SAS-Fucci cells.

## Discussion

In this study, we investigated the roles of IGFBP-3 in human TSCC SAS-Fucci cells originally derived from a TSCC with a highly malignant phenotype. RNA-Seq indicated that IGFBP-3 expression is involved in gene expression associated with cell migration and cell growth in SAS-Fucci cells. IGFBP-3 knockdown significantly reduced cell migration but also induced G_1_ arrest, which supports our RNA-Seq results.

Several studies have shown that cell-cycle progression is associated with cell migration. In primary mouse osteoblasts isolated from Fucci-expressing transgenic mice, cell migration velocity was highest in S/G_2_/M phases^42^. While both 2D and 3D models indicated no significant difference in cell migration between G1, early S, and S/G_2_/M phases in Fucci-expressing human melanoma cell lines^43^. In this study, we found that migratory potential is independent of cell-cycle position in both control and IGFBP-3 knockdown cells, which agrees with Haass et al.^43^. We also detected a time lag between the change in cell migration and cell-cycle distribution. We observed a reduction in migratory potential in IGFBP-3 knockdown cells at 48 h, which was followed by growth inhibition and G_1_ arrest. Intriguingly, we could not detect significant difference in migratory potential between cell-cycle phases even at 72 h when the cell-cycle distribution of IGFBP-3-knockdown cells was altered (Supplementary Fig. S4). Together, these findings suggest that IGFBP-3 may regulate migratory potential and cell growth independently, at least in SAS-Fucci cells. However, regarding the latter, cell line dependency is likely to exist (Supplementary Fig. S3).

The epithelial to mesenchymal transition (EMT) has been implicated in cell migration and invasion, in which TGF-β signaling plays a central role in cancer^44^. Moreover, IGFBP-3 has been shown to bind to and activate TGF-β receptor, thus leading to phosphorylation of Smad2 and Smad3 in breast cancer cells^9,10^. In human esophageal cells, IGFBP-3 induces EMT via TGF-β signaling activation^45^. In this study, GSEA analysis using our RNA-Seq data also revealed that IGFBP-3 expression level shows negative correlation with the EMT-related gene set: HALLMARK_EPITHELIAL_MESENCHYMAL_TRANSITION (NES =  − 1.66, *p* value = 0.0, FDR *q* value = 0.0077) (Supplementary Fig. S5a). However, any significance was not observed in the gene set related to TGF-β signaling: KEGG_TGF_BETA_SIGNALING_PATHWAY (NES =  − 1.06, *p* value = 0.929, FDR *q* value = 0.365) (Supplementary Fig. S5b), suggesting that IGFBP-3 contributes to EMT induction, but that this may be independent of TGF-β signaling in SAS-Fucci cells.

IGFBP-3 with a secretory signal peptide sequence is secreted, and it is important to determine the effect of secreted IGFBP-3 on cell migration. Notably, decreased migratory potential caused by IGFBP-3 depletion was not rescued by two different origins of rhIGFBP-3 proteins in SAS-Fucci cells, suggesting that intracellular IGFBP-3 is crucial for regulating cell migration. In contrast, the decreased cell migration observed among IGFBP-3 knockdown cells was restored to some extent by the application of hypoxic-conditioned medium. Hypoxia has been shown to upregulate the levels of IGFBP-3, IL-8, ODZ1, and CXCR4, in a HIF-1α dependent manner^32,51–54^. Indeed, hypoxic-conditioned medium contained a higher concentration of IGFBP-3 protein (~ 30 ng/mL) than that under normoxia but this level was much lower than the concentration of exogenously applied rhIGFBP-3 (100 ng/mL) used in this study. Hence, this result indicates that other factors induced by hypoxia may upregulate migratory potential in IGFBP-3 knockdown cells. Further studies are needed to identify the hypoxia-inducing factor that may promote cell migration in SAS-Fucci cells.

How does intracellular IGFBP-3 influence migratory potential? Many studies have demonstrated that activation of AKT and ERK also enhances migratory potential by upregulating the expression of genes related to cell migration^33–38^. We found that MEK and AKT inhibitors significantly reduced migratory potential (Fig. 5), which is consistent with past studies. Moreover, we observed that IGFBP-3 depletion inhibited ERK activity and was correlated with reduced migratory potential. In contrast, phosphorylated AKT levels were higher in IGFBP-3 knockdown cells compared with control cells. This result suggests both ERK and AKT activation are essential for promoting cell migration in SAS-Fucci cells. ERK activity is upregulated via activation of EGFR signaling^46^. Lin et al. demonstrated that IGFBP-3 interacts with EGFR and DNA-PK in breast cancer cells, which facilitates DNA damage repair^47^. Moreover, both intracellular and extracellular IGFBP-3 has been shown to stimulate EGFR signaling via upregulation of sphingosine kinase 1 and activation of sphingosine 1-phosphate receptor 1 and 3^48^. Given the functional association of IGFBP-3 with EGFR, IGFBP-3 may activate EGFR signaling, thereby enhancing cell migration. Upregulation of phosphorylated AKT may also result from the IGF-I-dependent pathway because the increased level of phosphorylated AKT was abrogated by IGF-IR inhibition (Supplementary Fig. S6). In general, IGFBP-3 binds to IGF-I and inhibits the IGF-IR signaling pathway^4,5^. Hence, IGFBP-3 depletion may increase free IGF-I levels, thereby activating IGF-IR signaling. Our previous study has demonstrated that IGF-I activates ERK in an EGFR-dependent manner, but that it activates AKT in an EGFR-independent manner^46^. This may explain the discrepancy between AKT and ERK activities caused by IGFBP-3 knockdown. Further studies are required to elucidate the roles of ERK and AKT in IGFBP-3 knockdown SAS-Fucci cells. Another possibility might be attributed to the direct interaction with β1 integrin^49^. IGFBP-3 upregulates the integrin activity and its downstream intracellular signaling pathways including the focal adhesion kinase (FAK) signaling pathway^49^. FAK signaling subsequently activates the MEK/ERK signaling cascade^50^. Thus, intracellular IGFBP-3 could enhance the ERK activity via EGFR and/or FAK signaling, which may contribute to cell migration and cell proliferation at least in SAS-Fucci cells.

To the best of our knowledge, we have demonstrated for the first time that IGFBP-3 is involved in gene expression related to cell migration and cell growth using RNA-Seq analysis. IGFBP-3 promotes tumor cell migration, at least partially, by modulating the ERK activity in SAS-Fucci cells, regardless of cell-cycle phase. IGFBP-3 therefore likely plays an important role in tumor metastasis and growth in TSCC, and identifying underlying mechanisms will facilitate the development of novel therapeutic strategies for targeting IGFBP-3, especially in endophytic-type TSCC.

## Methods

### Cell line

The SAS and HSC-3 cell lines, derived from human tongue squamous cell carcinoma and metastatic lymph nodes originated in human tongue squamous cell carcinoma, was obtained from the Health Science Research Resources Bank (Sendai, Japan) and from Dr S. Abe (Nihon University), respectively. SAS-Fucci cells and HSC3-Fucci cells were established as described previously^27,55^. Cells were cultured in DMEM containing high glucose (4500 mg/L) (Sigma-Aldrich, St. Louis, MO) with 100 units/mL penicillin and 100 μg/mL streptomycin, supplemented with 10% fetal bovine serum (FBS). Cells were incubated at 37 °C in a humidified 5% CO_2_ atmosphere.

### siRNA treatment

Cells were seeded 24 h before treatment with either *IGFBP3* Stealth RNAi™ siRNA (5′-UCCCAACUGUGACAAGAAGGGAUUU-3′; 5 nM; Invitrogen, Carlsbad, CA), *IGFBP3* Silencer Select siRNA (5′-CAUUCAAAGAUAAUCAUCAUU-3′; 5 nM; Ambion, Austin, TX, USA), or Stealth RNAi siRNA Negative Control Medium GC Duplex #2 (5 nM, Invitrogen) along with Lipofectamine RNAiMAX Transfection Reagent (Invitrogen). Cells were incubated for a further 48 or 72 h prior to experiment. Cells that were incubated for 72 h before experiments had their growth medium exchanged for fresh culture medium after 48 h.

### Drug treatment

Following siRNA treatment, fresh culture media were replaced and cells were treated with specific inhibitors of either IGF-IR (NVP-AEW541, 5 μM for 30 min; Novartis Pharmaceuticals, Basel, Switzerland), MEK (PD98059, 20 μM for 90 min; Wako Pure Chemical Industries, Osaka, Japan), or AKT (MK-2206, 5 μM for 30 min; Cayman Chemical, Ann Arbor, MI). Cells were also treated with 100 ng/mL recombinant human IGFBP-3 from either of two sources for either 90 min or 24 h before further experimental manipulation; the first was #675-B3, derived from NS0 mouse myeloma cell line (R&D Systems, Minneapolis, MN) and the second was ab280941, derived from HEK293T, a human embryonic kidney cell line (Abcam, Cambridge, UK).

Recombinant human IGFBP-3 proteins were reconstituted in a solution of 0.1% bovine serum albumin (BSA) in sterile PBS prior to use.

### Time-lapse imaging

A BIOREVO BZ-9000 fluorescence microscope (Keyence) was used for time-lapse imaging. Images were obtained at 30 min intervals. During imaging, cells were kept in an incubation chamber at 37 °C in a humidified atmosphere containing 95% air and 5% CO_2_ (Tokai Hit, Fujinomiya, Japan). Images were overlaid using BZ-X Analyzer (Keyence) and individual cells were tracked using ImageJ software^56^ (https://imagej.nih.gov/ij/) to quantify the distance traveled during the observation. Total distance traveled was divided by the observation time, which is presented as the average distance. The duration of the red phase was measured as the time elapsed between the beginning of the red phase to the end in each siRNA-treated cell.

### Quantitative real-time polymerase chain reaction (qPCR)

Following siRNA treatment, total RNA was isolated from cells using an RNeasy Mini Kit (Qiagen, Hilden, Germany). Subsequently, cDNA was synthesized from the RNA using SuperScript IV VILO Master Mix (Thermo Fisher Scientific, Waltham, MA). Oligonucleotide sequences for *IGFBP3* amplification were: forward primer, 5′-AAATGCTAGTGAGTCGGAGGA-3′ and reverse primer, 5′-CTCTACGGCAGGGACCATATT-3′. qPCR was performed with PowerUp SYBR Green Master Mix (Invitrogen) and analyzed using Applied Biosystems 7300 Real-Time PCR System (Thermo Fisher Scientific).

### Western blot analysis

Cells were lysed using Mammalian Protein Extraction Reagent (M-PER) (Thermo Fisher Scientific) containing protease inhibitor cocktail (cOmplete tablet; Roche, Mannheim, Germany) and phosphatase inhibitor cocktail (PhosSTOP; Roche). Equal amounts of proteins from cell lysates were separated by SDS-PAGE and transferred to PVDF membranes. Membranes were blocked using 5% ECL blocking agent (GE Healthcare, Chicago, IL) in Tris-buffered saline containing 0.05% Triton X-100. Proteins of interest were detected using primary antibodies against phosphorylated forms of AKT (Ser473; Cell Signaling Technology, Danvers, MA) and ERK1/2 (PROMEGA, Madison, WI), and against IGFBP-3 (B-5; Santa Cruz Biotechnology, Dallas, TX), AKT (Cell Signaling) and β-actin (clone C4; Millipore, Billerica, MA). Following incubation with primary antibodies, membranes were incubated with horseradish peroxidase-conjugated secondary antibodies (Santa Cruz Biotechnology). Detected proteins were visualized with ECL Western Blotting Detection reagents (GE Healthcare). Protein expression levels were quantified using ImageJ^56^. Active MAPK/ERK and p-AKT expression levels were normalized to β-actin and total AKT protein levels, respectively.

### Immunofluorescence staining

Cells were fixed with 4% paraformaldehyde in PBS for 15 min. After washing with PBS, cells were permeabilized by incubating in PBS-T for another 15 min. After blocking with 10% normal goat serum (Thermo Fisher Scientific) for 30 min, cells were incubated for 1 h with IGFBP-3 antibody (Santa Cruz Biotechnology). Next, cells were washed PBS-T before being incubated with Alexa Fluor 647-conjugated goat anti–mouse IgG secondary antibody (Invitrogen) and Hoechst 33342 (Invitrogen) for 30 min. Cells were subsequently imaged with a fluorescence microscope.

### Flow cytometric analysis

After siRNA treatment, cells were trypsinized and centrifuged. After washing the resulting cell pellets in PBS, cells were fixed with 4% paraformaldehyde in PBS for 15 min on ice. Fixed cells were washed in PBS before being permeabilized with 0.05% Triton X-100 in PBS. Permeabilized cells were blocked with 10% normal goat serum (Thermo Fisher Scientific) for 30 min and stained thereafter with Hoechst 33342 (Invitrogen) for 10 min. Cells were filtered through a nylon mesh to generate single-cell suspensions. Samples were analyzed by FACS Canto II (BD Bioscience, Franklin Lakes, NJ) and FlowJo software (BD Bioscience).

For the EdU Assay, we used the Click-iT EdU Alexa Fluor 647 Flow Cytometry Assay kit (Thermo Fisher Scientific). After siRNA treatment, cells were treated with 10 μM EdU and incubated for 1 h before being trypsinized. Cells were treated according to manufacturer’s protocol and analyzed with FACS Canto II and FlowJo software.

### Wound healing assay

Prior to time-lapse imaging, we dragged a 1250 μL pipette tip across a layer of confluent cells in the middle of a culture dish. The culture medium was replaced with fresh medium and images taken at two-hour intervals for 24 h under the same conditions as described above. Area of gap closure was measured using ImageJ software^56^. The closed area was calculated by subtracting the area at the indicated time from the area at 0 h. The percentage of closed area was normalized by the total area at 0 h in each group.

### Trans-well migration assay

Migration assays were performed with 24-well trans-well chambers (6.5 mm diameter, 8 μm pore size, Corning, Corning, NY). 8 × 10^4^ cells in 0.5% FBS-containing DMEM were seeded on the upper chamber while 10% FBS-containing DMEM was added in the lower well as a chemoattractant. After 18 h of incubation, cells that migrated were fixed with 4% paraformaldehyde and stained with crystal violet. The number of stained cells in in the entire field were counted and the percentage of migration was calculated by dividing the number of migrating cells in each group by the cell number in the control group.

### Cell proliferation assay

One thousand five hundred cells were seeded per well of a 96-well culture plate and incubated for 24 h before siRNA treatment as described. CCK-8 reagent (10 μL) from Cell Counting Kit 8 (Dojindo, Kumamoto, Japan) was added to each well and cells were incubated for an additional 1.5 h at 37 °C before absorbance was measured at 450 nm with a Multiscan FC Microplate Reader. Relative absorbance was calculated by subtracting the absorbance measured in cell-free medium from that in medium containing either IGFBP-3 knockdown or control cells.

### Hypoxic treatment

SAS-Fucci cells were seeded 24 h prior to hypoxic treatment. Hypoxic treatment was achieved by sealing culture dishes in an air-tight container with Anaeropack-Anaero 5% system (Mitsubishi Gas Chemical, Tokyo, Japan). The container was incubated at 37 °C for 24 h before the culture dishes were removed from the container. Hypoxic-conditioned medium was collected from the dishes and centrifuged to remove cells and cell debris. Normoxic-conditioned medium was obtained from dishes of cells incubated under normoxic conditions for the same duration. The supernatant was later used for either ELISA or cell treatment.

### ELISA

Human IGFBP-3 ELISA Kit from Abcam (ab211652) was used to measure the concentration of IGFBP-3 in culture medium. Culture medium was obtained from dishes and centrifuged to remove cells and debris from the medium. Samples were subsequently treated as required by the ELISA kit. Absorbance was measured with a Multiscan FC Microplate Reader, and sample concentrations were calculated from standard curve readings.

### RNA sequencing

Total RNA was extracted using the RNeasy Mini Kit (Qiagen). The RNA-Seq was performed by Azenta Japan (Tokyo, Japan) using a DNBSEQ-G400 (MGI Tech, Shenzhen, China). Hisat2 (version 2.0.1) was used to align the data to reference genome. Gene set enrichment analysis and gene ontology analysis were performed using the software GSEA (version 4.1.0; https://www.gsea-msigdb.org/gsea/index.jsp) and DAVID (https://david.ncifcrf.gov/tools.jsp), respectively.

### Statistical analysis

Differences in *IGFBP3* mRNA level were determined by qPCR, migratory potentials by cell tracking analysis and trans-well migration assay, the concentration of secreted IGFBP-3 by ELISA, and cell-cycle distribution and EdU incorporation by FACS analysis, were analyzed using either a two-tailed Student’s t-test, one-way ANOVA with Tukey’s multiple comparison test, or Kruskal–Wallis test with Dunn’s multiple comparisons test. The significance of differences in wound healing and in cell proliferation was determined using two-way ANOVA and Sidak’s multiple comparisons test. Kaplan–Meier survival analysis was performed on the TCGA dataset and the results evaluated using the log-rank test. Statistical analyses were performed using GraphPad Prism software (GraphPad Software, San Diego, CA); *p* values < 0.05 were considered statistically significant.

## Supplementary Information

Supplementary Figures.

## Data Availability

RNA-Seq data generated and/or analyzed during the current study are available in the Gene Expression Omnibus (GEO) repository, Accession Number GSE205275. The other datasets either generated and analyzed or just analyzed in this study are available from the corresponding author upon reasonable request.
