# Supplementary material for: Roles of IGFBP-3 in cell migration and growth in an endophytic tongue squamous cell carcinoma cell line
Source: Sci Rep. 2022 Jul 7;12:11503. doi: 10.1038/s41598-022-15737-y (PMC9262895; doi:10.1038/s41598-022-15737-y)
Supplement: Supplementary file 1 — Supplementary Figures. [file 41598_2022_15737_MOESM1_ESM.pdf]

Supplementary Information

Roles of IGFBP-3 in cell migration and growth in an endophytic tongue squamous cell carcinoma cell line

Esther Feng Ying Ng, Atsushi Kaida*, Hitomi Nojima,

Masahiko Miura*

Department of Oral Radiation Oncology, Graduate School of

Medical and Dental Sciences, Tokyo Medical & Dental

University, Japan

Supplementary Figure S1 relevant to Figure 2

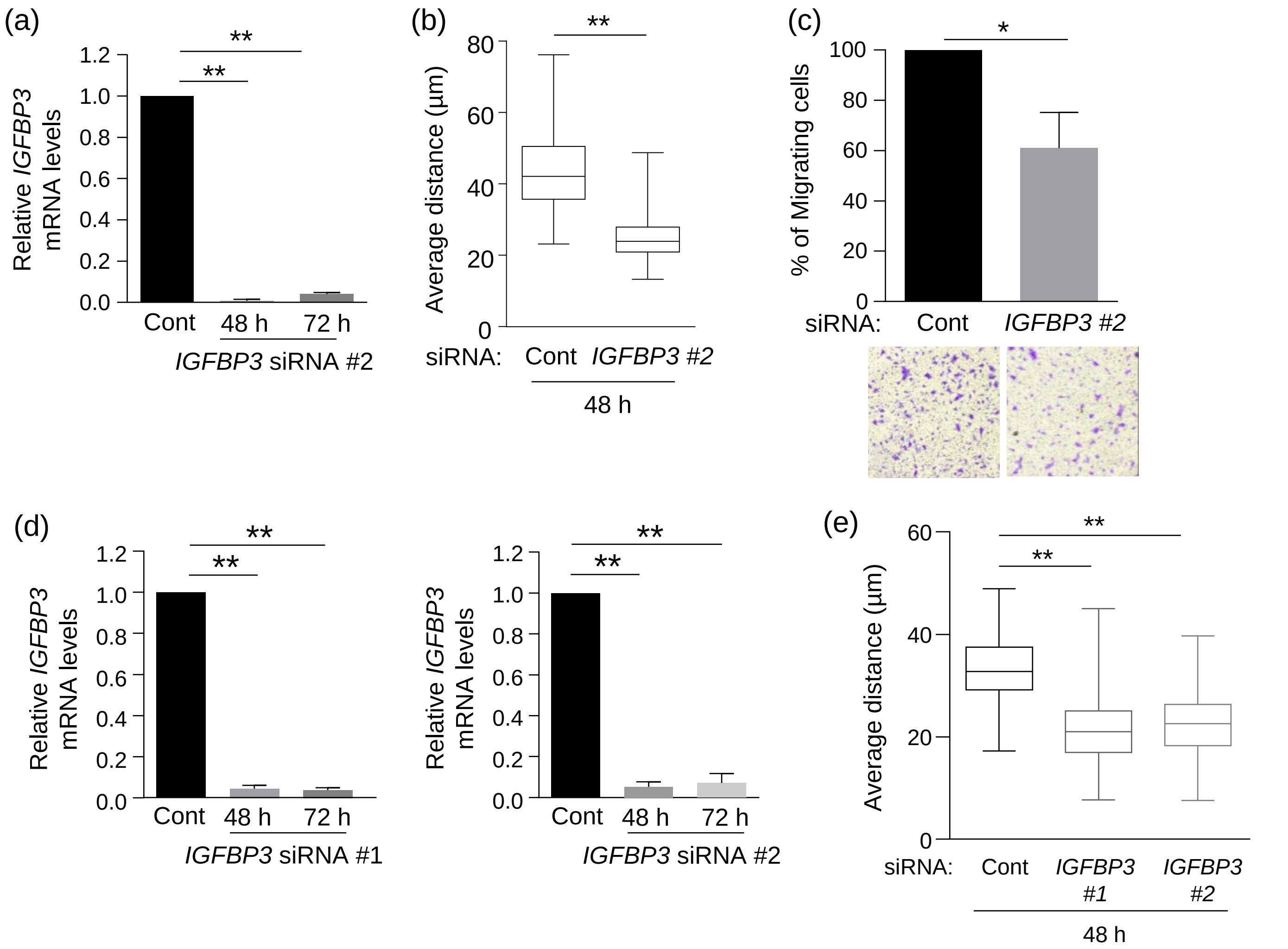

Supplementary figure S1. Reduced cell migration in IGFBP-3 knockdown cells. (a) *IGFBP3* mRNA levels determined by qPCR. SAS-Fucci cells were transfected with either non-targeted control (Cont) or IGFBP3 siRNA #2 (48 or 72 h). Total mRNA was extracted either 48 or 72 h after siRNA treatment. Means ± S.D. (n = 3 independent experiments). (b) Average distance travelled by SAS-Fucci cells during a 10 h period 48 h after transfection with either non-targeted control (Cont) or *IGFBP3* siRNA #2. This distance is represented as a box and whisker plot showing outliers, distribution intervals, interquartile range (box), and median. Cell numbers in each group ranged from 159–200. Two independent experiments were performed, and representatives are shown here. (c) Quantitative analysis (top) and representative images (bottom) of trans-well migration assays using IGFBP-3-knockdown (*IGFBP3* #2) and non-targeted control (Cont) SAS-Fucci cells (8×10^4 cells). Migrating cells in the entire field were counted 18 h later. Means ± S.D. (n = 3 independent experiments). (d) *IGFBP3* mRNA levels determined by qPCR. HSC3-Fucci cells were transfected with either *IGFBP3* siRNA #1 (left) and #2 (right) or non-targeted control siRNA (Cont) (48 or 72 h). Total mRNA was extracted either 48 or 72 h after siRNA treatment. Means ± S.D. (n = 3 independent experiments). (e) Quantification of average distance during 10 h using HSC3-Fucci cells 48 h after transfection with either *IGFBP3* siRNA #1 and #2 or non-targeted control siRNA (Cont). Each average distance is represented as a box and whisker plot showing outliers, distribution intervals, interquartile range, and median. Cell numbers in each group were 100. Two independent experiments were performed, and representatives are shown. *$p < 0.05$, **$p < 0.01$; one-way ANOVA with Sidak's multiple comparisons test (a), Mann-Whiney U-test (b), two-tailed Student's *t*-test (c), two-way ANOVA with Sidak's multiple comparisons test (d), Kruskal-Wallis test with Dunn's multiple comparisons test (e).

Supplementary Figure S2 relevant to Figure 4

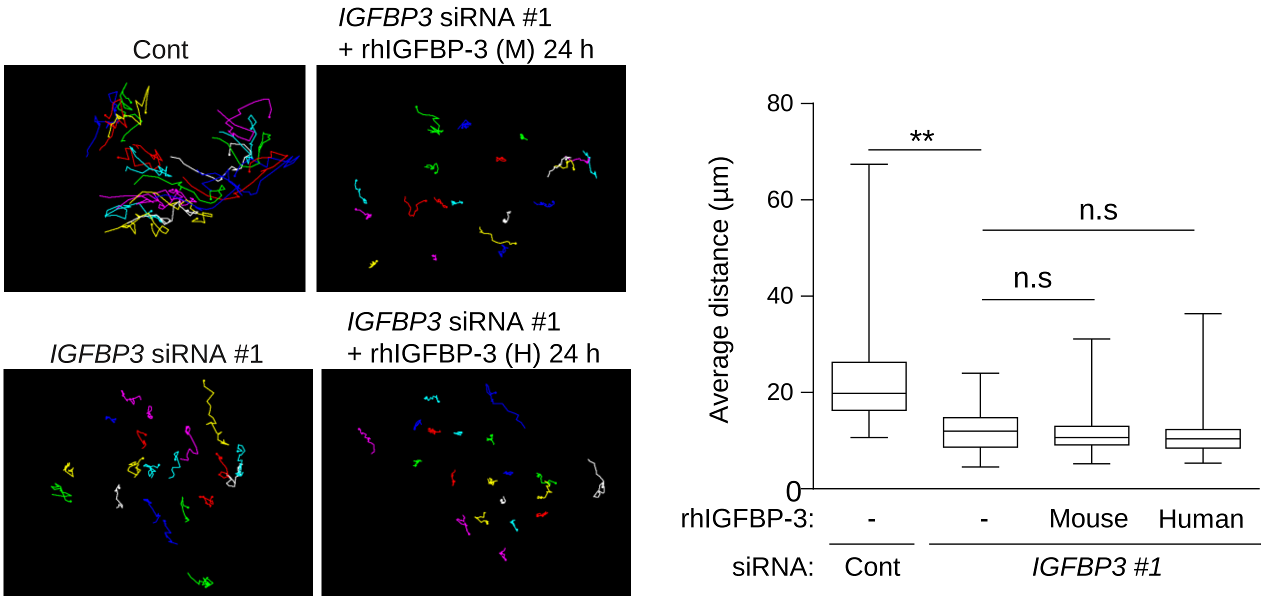

Supplementary figure S2. Non-significant effect of prolonged treatment with exogenous IGFBP-3 on cell migration. Representative images of individual cell tracks (left) and average distance traveled (right) by control (Cont) and IGFBP-3 knockdown (*IGFBP3* siRNA #1) cells with or without recombinant human IGFBP-3 (rhIGFBP-3) derived from either mouse or human cells. rhIGFBP-3 was added 48 h after siRNA treatment; cells were pre-treated with siRNA for 24 h before time-lapse imaging. Each colored line indicates the path taken by individual cell tracks during the observation period. Each average distance is represented as a box and whisker plot showing outliers, distribution intervals, interquartile range (box), and median. Cell numbers in each group ranged from 80–100. Either two or three independent experiments were performed, and representatives are shown here. *p < 0.05, **p < 0.01; Kruskal-Wallis test with Dunn's multiple comparisons test.

Supplementary Figure S3 relevant to Figure 6

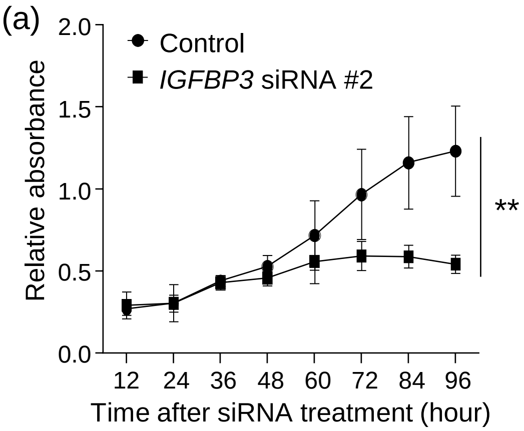

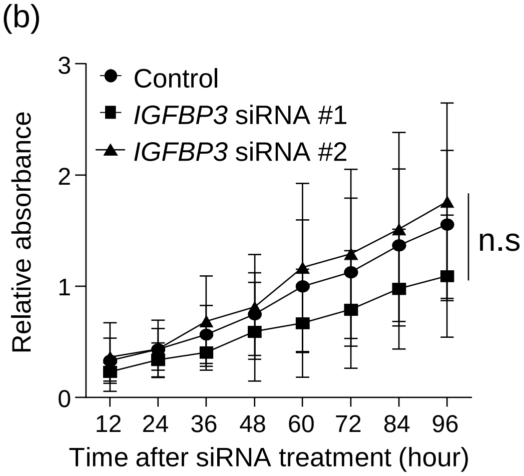

Supplementary figure S3. Reduced cell proliferation in IGFBP-3 knockdown SAS-Fucci cells but not HSC3-Fucci cells. (a and b) Quantitative analysis of cell proliferation assay using IGFBP-3 knockdown (*IGFBP3* siRNA #1 or #2) and non-targeted control (Cont) SAS-Fucci cells (a) and HSC3-Fucci cells (b). The Cell Counting Kit-8 assay was performed each time after siRNA treatment and relative absorbance was plotted. Means ± S.D. (n = 3 independent experiments). ** $p < 0.01$, two-way ANOVA with Sidak's multiple comparisons test (a, b).

Supplementary Figure S4 relevant to Discussion

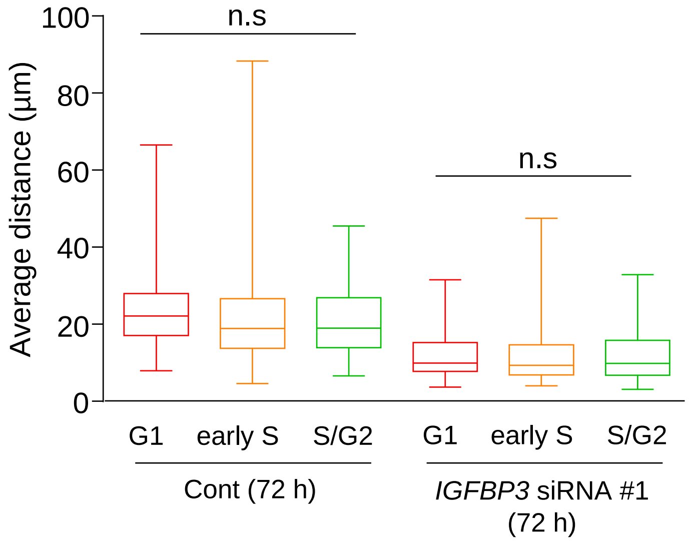

Supplementary figure S4. No difference in migratory potential between cell-cycle phases 72 h after siRNA treatment. Quantification of average distance of cells in each cell cycle phase using control (Cont) and IGFBP-3 knockdown (*IGFBP3* siRNA #1) cells at 72 h. Cells were classified as being in either G_1, early S, or S/G_2 according to fluorescent color and cell morphology. Each average distance is represented as a box and whisker plot showing outliers, distribution intervals, interquartile range (box), and median. The number of cells in each group ranged from 88–170 cells. Three independent experiments were performed, and representatives are shown here. n.s. (not significant); Kruskal-Wallis test with Dunn’s multiple comparison test.

Supplementary Figure S5 relevant to Discussion

(a)

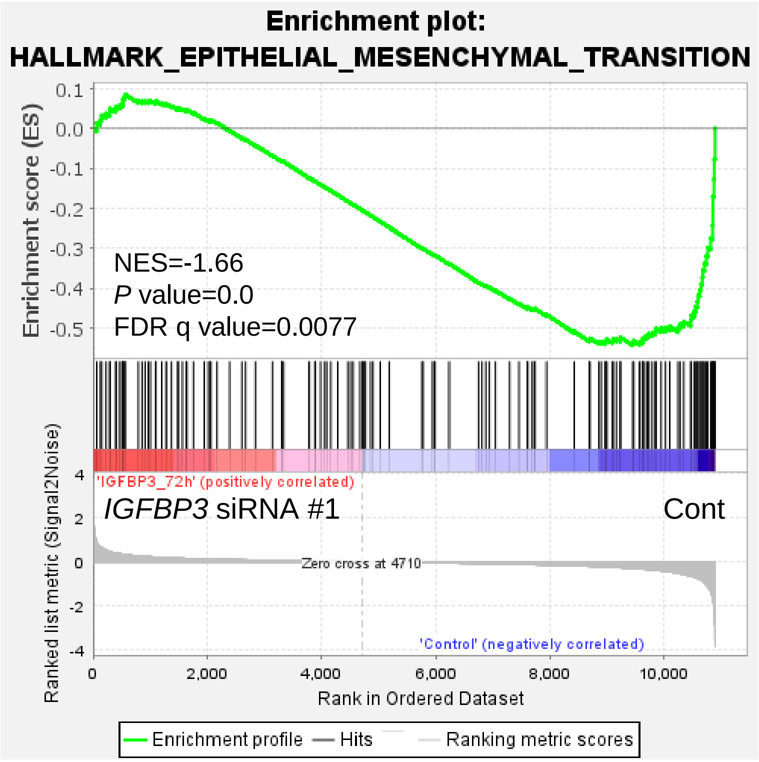

(b)

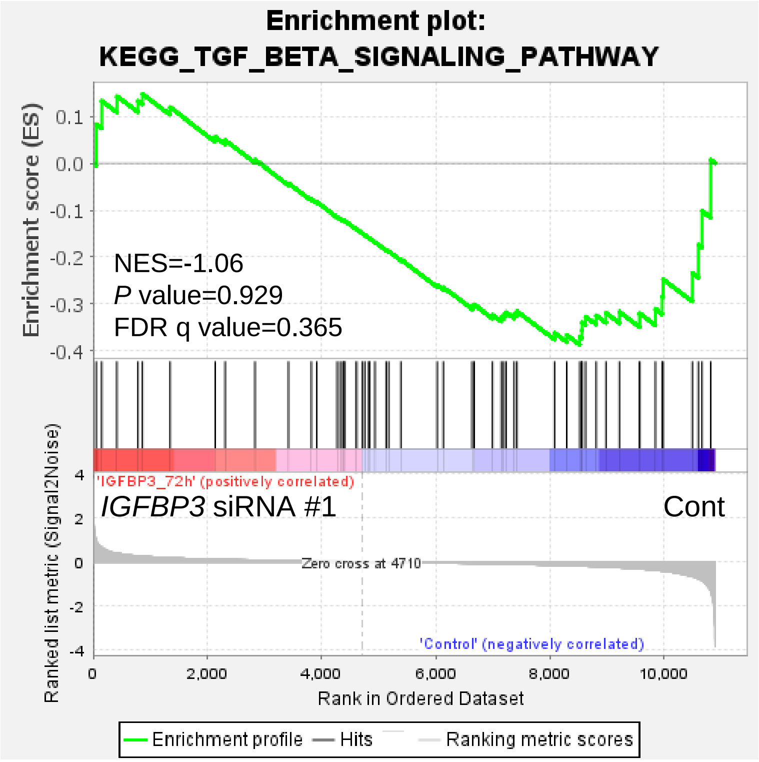

Supplementary figure S5. Correlation of IGFBP-3 expression with genes related to EMT but not TGF-β signaling. **(a and b)** GSEA of EMT (a) and TGF-β signaling (b) signatures between control (Cont) and IGFBP-3 knockdown (*IGFBP3* siRNA #1) cells. NES, normalized enrichment score; FDR, false discovery rate.

Supplementary Figure S6 relevant to Discussion

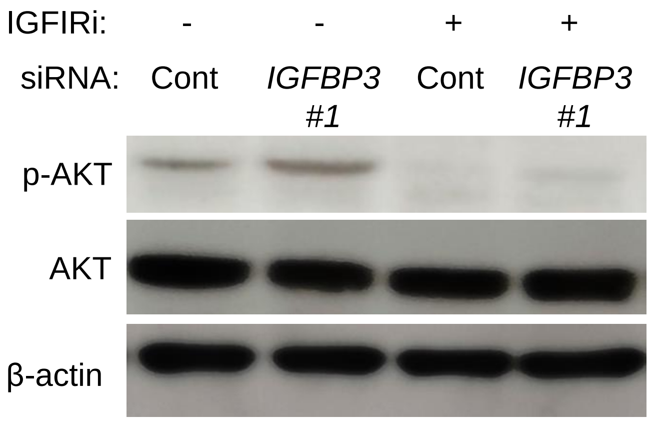

Supplementary figure S6. Abrogation of increased levels of phosphorylated AKT by IGFBP-3 depletion and IGF-IR inhibition. Western blotting for phosphorylated AKT (p-AKT), AKT, and β-actin in control (Cont) and IGFBP-3 knockdown (*IGFBP3* #1) cells in either the presence or absence of IGF-IR inhibitor (NVP-AEW541; IGFIRi, 5 μM). Cells were incubated with siRNA for 48 h. The inhibitor was added 30 min before cell lysates were extracted. Three independent experiments were performed, and representatives are shown. Uncropped blots are presented in Supplementary Fig. S7.

Supplementary Figure S7 relevant to Discussion

Fig.1c

IGFBP-3

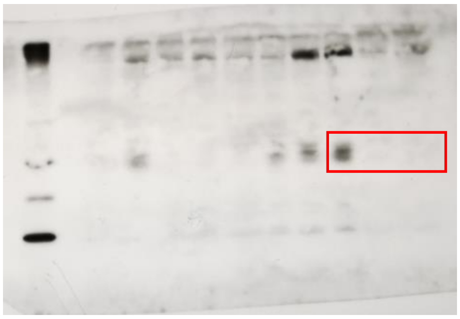

β-actin

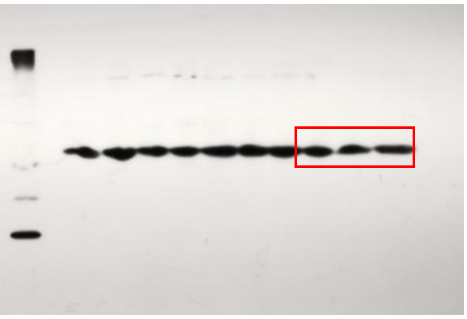

Fig.5a

Active MAPK/ERK

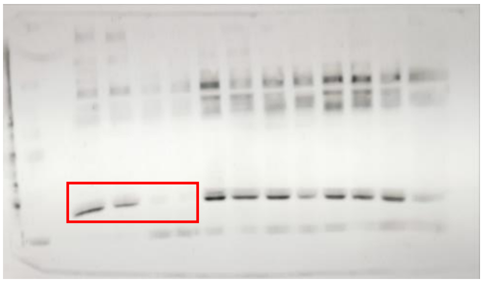

β-actin

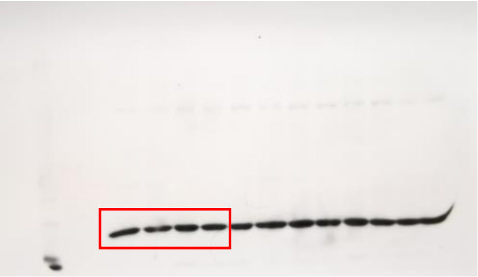

Fig.5b

p-AKT

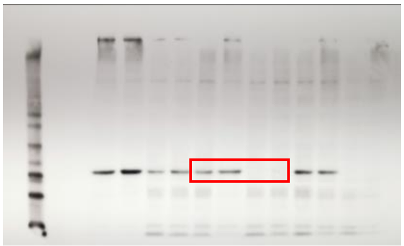

AKT

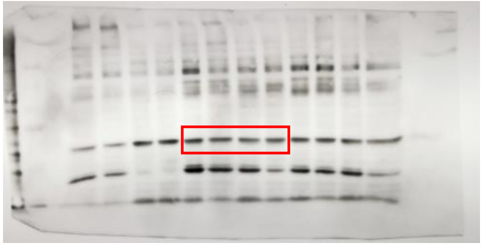

β-actin

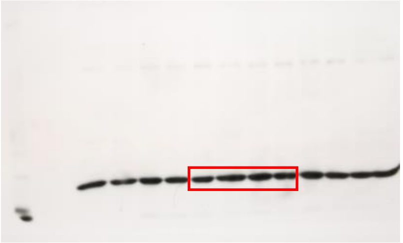

Supplementary fig.S6

p-AKT

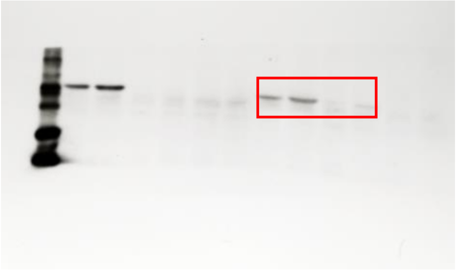

AKT

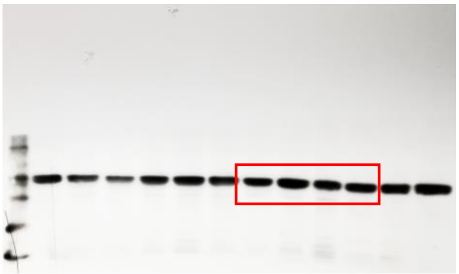

β-actin

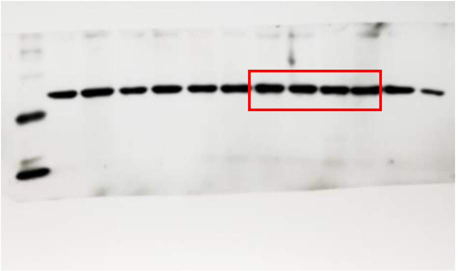

Supplementary figure S7. Uncropped western blot images in Fig. 1, Fig. 5, and Supplementary Fig. S6.
